# Application of Antigen Cross-Presentation Research into Patient Care

**DOI:** 10.3389/fimmu.2014.00287

**Published:** 2014-06-17

**Authors:** Thijs W. H. Flinsenberg, Marianne Boes

**Affiliations:** ^1^Laboratory of Translational Immunology, Department of Pediatrics, University Medical Center Utrecht, Utrecht, Netherlands

**Keywords:** immunotherapy, dendritic cell, cancer vaccines, clinical trial, virus infection, autoimmunity, T-cells

Dendritic cell (DC)-based cellular immunotherapy is being explored as a treatment modality for several malignancies, for viral diseases and auto-immune disorders. More than four decades of pre-clinical research on DC biology has cemented a strong foundation for clinical application of DC-based clinical trials, which already have been performed since the 1990s. Although sometimes met with limited patient success, clinical trials do yield better understanding of the requirements for optimal DC-based therapy. Recent advancements in the understanding of human DC biology and subset characteristics now give rise to ample opportunities to explore for a next generation of DC-based immunotherapy. This Research Topic is focused on articles that can help understanding the biology involved in DC antigen presentation, for future DC-based immunotherapy.

Dendritic cells are professional antigen presenting cells (APCs) that are particularly well endowed to elicit adaptive immune responses, via the presentation and cross-presentation of antigen-derived peptide/MHC complexes to T-lymphocytes. These processes decide how the host interacts with its environment, and therefore can be a target for pathogen interruption. van Montfoort et al. (1) provide an overview of cross-presentation features and describe how the study of various viral pathogens can elucidate anti-viral immune strategies. They further describe how DC maturation is crucial in immunity against viruses and how viruses may dampen this response to their own advantage. Understanding these presentation pathways is pivotal to develop effective DC-based immunotherapy.

Dendritic cell-based immunotherapy comes in two flavors. Either DCs are cultured and manipulated *ex vivo* before infusion, or endogenous DCs can be targeted *in vivo*. Concerning the latter approach, local administration of long peptides has proved effective in several diseases giving opportunity for further exploration. Both Delamarre and Cohn (2) and Rosendahl Huber et al. (3) discuss the requirements for improved antigen presentation, providing considerations on CD4^+^ and CD8^+^ activation, choice of antigen, and desired adjuvants. Delivering antigen to DCs is a next hurdle to take.

Regarding antigen delivery, it matters to engage responsive receptors, for these receptors can decide the intracellular pathway of antigen routing and enzymatic processing. Antigens are thereby directed toward assembly into peptide/MHC class I (cross-presentation) or peptide/MHC class II complexes, and induction of immunity or tolerance. Fehres et al. (4) describe the biology of receptor-mediated uptake in the context of antigen presentation, with special emphasis on C-type Lectin receptors. They further discuss the possibilities to formulate antigen in order to provide receptor-directed antigen delivery. Another import route of uptake involves the family of Fc receptors, which is discussed by Platzer et al. (5). Here, the role of this receptor family is highlighted in antigen presentation with emphasis on the opposing roles of activating and inhibiting Fc Receptor isoforms. Furthermore, they underscore that mechanisms of antigen presentation in mice are not always identical to the human pathways. Thus, the need for more research on human DC biology is warranted, for DC-vaccination strategies are still heavily based on mouse-biology.

When designing a DC-based immunotherapy, it is relevant to consider the subtype of DCs that one aims for. Boltjes and van Wijk (6) present an overview of all phagocyte subsets that are present throughout the human body in steady-state and under inflammatory conditions. They also emphasize the differences between mouse and human cells, and review cell types that should be considered for immunotherapy. Until recently, monocyte-derived DCs (MoDCs) were used mostly in DC-therapy, for their relative ease to culture in large quantities *ex vivo*. But while MoDCs can be found in human tissue under inflamed conditions, other DC subsets are more prevalent overall and may be more specifically endowed at stimulation of particular T-cell subsets, to be explored in immunotherapy. One subset that was suggested to be superior in CD8^+^ T-cell priming is the recently identified BDCA-3^+^ (CD141^+^) DC, characterized by CLEC9A and XCR1 expression. Tullett et al. (7) highlight recent findings explaining why these cells are effective at CD8^+^ T-cell priming and discuss *in vivo* antigen targeting toward these DCs. Wimmers et al. (8) also describe the use of naturally circulating mDCs and pDCs for DC-based immunotherapy. They discuss the division of labor between pDCs and mDCs and the clinical trials that are being performed using these subsets. Interestingly, they highlight that mDCs and pDCs work in synergy, supporting each other to enhance the effector phase of the adaptive immune response. Based on this observation, a next step in DC-based vaccination should include a cocktail of mDCs and pDCs, or *in vivo* antigen targeting to both subtypes.

Dendritic cells are often called “master regulators” of the immune response. Besides firing up immune reactions, DCs play an equally important role in the maintenance of peripheral tolerance, for example by dampening specific T-cell responses or by inducing regulatory T-cell subsets. Loss of tolerance is of pivotal importance in auto-immune diseases as described by Hopp et al. (9). Their review concerns the presentation of self-antigen, which they discuss in the context of mechanisms in tolerance induction, DC maturation status, DC uptake and processing mechanisms, and tolerance-associated intracellular signaling pathways. The regulation of DC function is also controlled by metabolic pathways, as described by Everts and Pearce (10). Recent advancements concerning regulation of DC metabolism include the identification of key-proteins like PI3K, Akt, and mTOR in DC function. The awareness that manipulation of DC metabolic pathways changes DC function should be explored for designing DC-based cellular therapy, especially since it may give opportunity to steer toward more immunogenic or tolerogenic consequences. This could be of upmost importance in the setting of auto-immune diseases, anti-cancer, or graft-versus-host therapy.

Plantinga et al. (11) finally discuss recent developments in DC-therapy in the setting of allogeneic–hematopoietic cell transplantations (HCT). Such transplantations are considered a last-resort treatment for several malignancies of hematological origin. DCs grown from the same donor background as the HCT are now being explored for their potency to prevent cancer relapses early after allogeneic HCT. The various considerations for such DC vaccinations are discussed, such as the stem cell source, type of tumor antigen, and vaccination strategy.

The breadth and quality of the work discussed in this Research Topic underscores the strong translational push of DC research toward clinical settings (Figure 1). Immunotherapy is now being incorporated into standard cancer care, with antibody-based treatments currently being at more advanced stages than cellular therapies. The abundance of currently ongoing DC-based cellular immunotherapy trials should benefit patient care in the near future, as the roots for translational success are implanted in well-established pre-clinical research settings.

**Figure 1:**
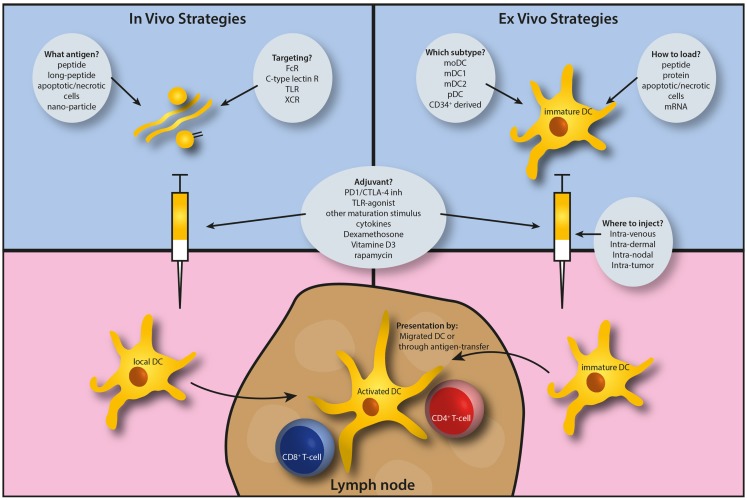
**Schematic outline of the considerations to apply antigen cross-presentation research to the clinic, most readily by dendritic cell-based immunotherapy**.

## Conflict of Interest Statement

The authors declare that the research was conducted in the absence of any commercial or financial relationships that could be construed as a potential conflict of interest.
